# Takotsubo Cardiomyopathy Associated With Checkpoint Inhibitor Therapy: Endomyocardial Biopsy Provides Pathological Insights to Dual Diseases

**DOI:** 10.1016/j.jaccao.2021.02.005

**Published:** 2021-06-15

**Authors:** Michael Serzan, Suthee Rapisuwon, Jayashree Krishnan, Ian C. Chang, Ana Barac

**Affiliations:** a Division of Oncology, Department of Medicine, Lombardi Comprehensive Cancer Center, Medstar Georgetown University Hospital, Washington, DC, USA; b Department of Pathology, Medstar Washington Hospital Center, Washington, DC, USA; c Division of Cardiology, Department of Medicine, MedStar Heart and Vascular Institute, Medstar Washington Hospital Center, Washington, DC, USA

**Keywords:** histopathology, imaging, immune checkpoint inhibitors, immunotherapy, Takotsubo cardiomyopathy, ICI, immune checkpoint inhibitor, irAE, immune-related adverse event, TTE, transthoracic echocardiogram

**Figure:**
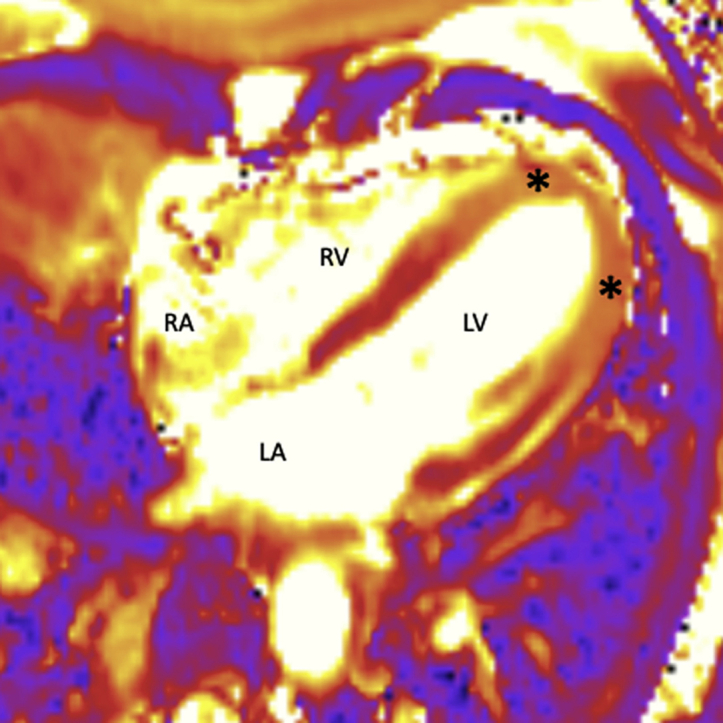

Immune checkpoint inhibitors (ICIs) are monoclonal antibodies to T cell regulatory pathways mediated by cytotoxic T lymphocyte-associated antigen-4 (CTLA-4), programmed cell death-1 (PD-1), or programmed cell death-ligand 1 (PD-L1). Therapeutic inhibition of these pathways leads to restitution of the endogenous antitumor response through a wide variety of pathways, including direct T cell cytotoxicity, impaired T_reg_ cell function, and increased inflammatory cytokines. ICIs are often associated with collateral organ inflammation termed immune-related adverse events (irAEs), commonly causing dermatitis, colitis, pneumonitis, hepatitis, and endocrinopathies (1). Cardiovascular irAEs have been characterized as myocarditis, pericardial disease, vasculitis, Takotsubo cardiomyopathy, arrhythmias, and destabilization of atherosclerotic disease (2). Although incidence of ICI myocarditis is uncommon, estimated at 0.04% to 1.14%, these irAEs can be severe with mortality estimated at 25% to 50% (2).

Takotsubo cardiomyopathy is a syndrome of acute, transient regional left ventricular (LV) dysfunction in the absence of obstructive coronary artery disease. Although the precise pathophysiology is unknown, chronic inflammation is hypothesized to account for long-term complications, such as heart failure, arrhythmias, thrombosis, and structural heart disease (3). Clinically, Takotsubo cardiomyopathy has been described as a mimicker of acute coronary syndrome, and exclusion of ischemia is part of the diagnostic algorithm. Less is known about the diagnostic approach in patients presenting with concern for myocarditis. Herein, we describe a patient on adjuvant combined anti-CTLA-4 and anti-PD-1 therapy who presented with acute severe LV dysfunction and regional wall motion abnormalities.

## Case Report

A 66-year-old woman, a former 30 pack-year smoker, was diagnosed with T2aN0M0 high-risk left choroidal melanoma as defined by a class 2 gene expression profile. Pre-treatment transthoracic echocardiography (TTE) revealed normal LV size and an ejection fraction of 60%. The patient underwent primary I-125 plaque brachytherapy with staging computed tomography (CT) scan showing no evidence of metastatic disease. She participated in a phase II adjuvant immunotherapy clinical trial (HCRN MEL17-309; NCT03528408) with ipilimumab [anti–CTLA-4] 1 mg/kg every 6 weeks and nivolumab [anti–PD-1] 240 mg every 2 weeks to prevent recurrence of high-risk ocular melanoma.

Approximately 16 weeks into adjuvant treatment (3 doses ipilimumab and 7 doses nivolumab), the patient presented to an outside hospital with dyspnea on exertion and generalized pain. She reported no chest pain, palpitations, syncope, fevers, cough, or sputum production. The electrocardiogram (ECG) revealed sinus tachycardia with new inferolateral T-wave inversions concerning for ischemia. TTE showed apical akinesis and hyperdynamic basal LV segments. CT angiography of the chest showed no evidence of pulmonary embolism, with bilateral mild patchy parenchymal opacities in the lower lobes, compatible with infectious or inflammatory changes, and extensive coronary artery calcifications. A coronavirus disease-2019 test was negative. Laboratory evaluation revealed an elevated high-sensitivity cardiac troponin I level of 539 ng/l (normal 0 to 34 ng/l), which peaked at 900 ng/l. Creatinine kinase and natriuretic peptides were not measured. The patient was transferred to our institution for coronary catheterization for suspected non–ST-segment elevation myocardial infarction.

**Figure 1:**
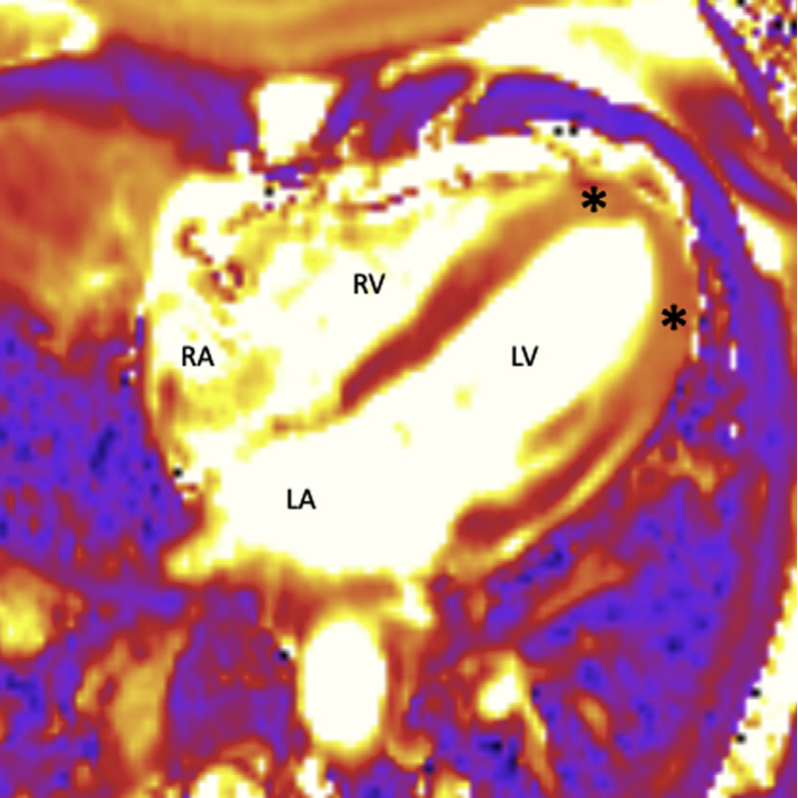
Cardiac Magnetic Resonance Imaging T_2_-weighted dark blood images **(asterisks)** show increased signal intensity in the left ventricular (LV) apical segments indicating presence of myocardial edema. LA = left atrium; RA = right atrium; RV = right ventricle.

On arrival, her vital signs were stable (temperature 37.1°C, blood pressure 114/73 mm Hg, heart rate 94 beats/min, O_2_ saturation 96% on room air), lung sounds were normal, and there was no evidence of jugular venous distention or lower extremity edema on examination. Left heart catherization revealed nonobstructive coronary artery atherosclerosis. Left ventriculography confirmed LV apical akinesis. Cardiac magnetic resonance imaging showed severe hypokinesis of the apical segments with an increased signal intensity on T_2_-weighted dark blood images and T_2_- and T_1_-weighted parametric maps (Figure 1). A very soft diffuse signal without focal areas of enhancement was seen on late gadolinium-enhanced images. These imaging characteristics, together with relative hypercontractility of the basal segments, were suggestive of Takotsubo cardiomyopathy; however, ICI-related myocarditis could not be excluded.

**Figure 2:**
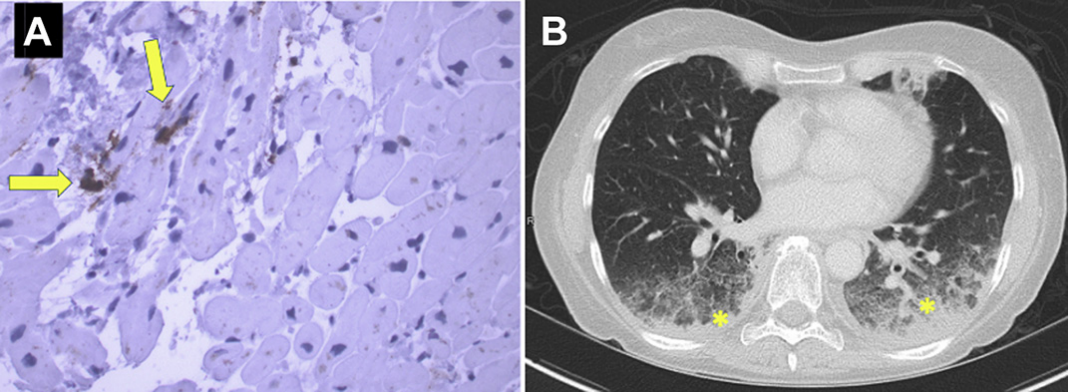
Endomyocardial Biopsy and Chest Computed Tomography **(A)** Endomyocardial biopsy shows focal fibrosis without malignancy or myocarditis. Immunostaining for CD68 shows very rare macrophages **(yellow arrows)**. **(B)** Chest computed tomography shows moderate consolidation in the bilateral posterior lower lobes with adjacent ground glass opacities **(yellow asterisks).**

Right ventricular catherization was therefore performed and 4 endomyocardial biopsy samples were obtained from the distal right ventricular septal wall. Pathology showed focal fibrosis without evidence of lymphocytic infiltrate and rare CD3 and CD68^+^ cells (Figure 2A). Immunohistochemistry showed no staining for PD-L1 expression. The World Health organization (WHO) criteria for a pathological diagnosis of myocarditis were not met: there was no histological evidence of myocardial inflammatory infiltrate or nonischemic myocyte degeneration/necrosis, and there was absence of immunohistochemical criteria (abnormal inflammatory infiltrate defined as ≥14 leukocytes/mm^2^ with the presence of CD-3–positive T-lymphocytes ≥7 cells/mm) (4). Therefore, immunosuppressive therapy was not initiated and the patient was started on metoprolol for symptomatic management of Takotsubo cardiomyopathy.

Three days later and 1 week after the initial presentation, the patient presented with worsening dyspnea on exertion, diffuse myalgias, and low-grade fevers. Her temperature was 38.8°C, blood pressure 128/70 mm Hg, heart rate 99 beats/min (normal sinus rhythm), O_2_ saturation 92% on room air. ECG showed normal sinus rhythm with anterolateral T-wave inversion and laboratory evaluation showed a down-trending high-sensitivity cardiac troponin I 57 ng/l. Repeat TTE revealed an LV ejection fraction 65% to 70% with resolution of apical akinesis. Her WBC was 16 k/μl (normal 4 to 11 k/μl), C-reactive protein 226 mg/l (normal 0 to 10 mg/l), erythrocyte sedimentation rate 125 mm/h (normal 0 to 22 mm/h), creatinine kinase 111 U/l (normal 34 to 145 U/l), and B-type natriuretic peptide 262 pg/ml (normal 0 to 99 pg/ml). Infectious work-up was negative, and CT scan of the chest showed worsening moderate bibasilar consolidation with ground glass opacities without pleural effusions (Figure 2B). The patient was diagnosed with Grade 3 ICI pneumonitis evidenced by symptomatic dyspnea on exertion, new-onset hypoxia, and radiographic ground glass opacities in >50% of lung parenchyma. Immunosuppressive therapy was initiated with solumedrol 1 mg/kg twice daily resulting in rapid improvement within 3 days. She was discharged on prednisone 60 mg daily with plan for gradual taper over 6 weeks. CT scan of the chest 6 weeks later showed near-resolution of ICI pneumonitis, and cardiac magnetic resonance imaging 3 months later showed significant improvement in cardiac function with normalization of contractility in the apical segments and resolution of edema on T_2_-weighted images.

## Discussion

This case highlights the pathophysiological complexity of Takotsubo cardiomyopathy and cardiovascular irAEs on ICI therapy, which may lead to clinically relevant challenges in diagnosis. In clinical scenarios with overlapping characteristics, endomyocardial biopsy represents an important tool to exclude ICI-related myocarditis.

Takotsubo cardiomyopathy has been described in patients with advanced malignancies with potential triggers, including emotional disturbances, cancer surgery, radiation therapy, and cytotoxic chemotherapies (5). Although the pathophysiology of Takotsubo cardiomyopathy remains unknown, several hypotheses have been proposed, including coronary vasospasm, microvascular dysfunction, and excessive catecholamine response to physical or psychological stress (5). More recently, Wilson et al. (3) have proposed that inflammation has an essential role in Takotsubo cardiomyopathy utilizing a rodent model of catecholamine-induced cardiomyopathy (3). After catecholamine induction, early infiltration of neutrophils and a relative increase in proinflammatory M1 (CD68^+^) macrophages were observed. A predominance of proinflammatory M1 macrophages was also seen in postmortem myocardial tissue from a patient with Takotsubo cardiomyopathy. Similarly, immunostaining of our patient’s endomyocardial biopsy specimen showed CD68^+^ M1 macrophages, suggesting a role for inflammation in Takotsubo cardiomyopathy and supporting the hypothesis that proinflammatory CD68^+^ M1 macrophages are a possible biomarker of disease.

irAEs are common with grade 3 to 4 toxicity rates of approximately 30% on anti-CTLA-4, 10% on anti-PD-1/PD-L1, and 60% on combination ICI therapy (1). Although the precise mechanisms of irAEs continue to be investigated, several key pathways have been delineated including dysfunction in T_reg_ cells (leading to cytokine release, effector T cell activation, and loss of self-tolerance), B-cell–mediated autoantibody production, cross-reactivity between antitumor T cells and homologous antigens on healthy tissues, and complement-mediated tissue damage from direct binding of antibodies to CTLA-4 and PD-L1 expressed on normal tissue (1). Pathology reports of patients treated with ICIs and meeting the WHO criteria for myocarditis have also described clonal CD3+ T cell infiltration, autoantibody immunoglobulin G deposition, and PD-L1 expression (2). Johnson et al. (6) report 2 patients treated with ipilimumab and nivolumab who were diagnosed with fulminant myocarditis with dense infiltration of clonal T cells. Although they promptly received corticosteroids, both patients died from cardiac arrest reflecting the uncommon yet often sometime fulminant irAE outcomes.

The clinical diagnosis of ICI-related myocarditis can be challenging, and consideration of the clinical syndrome, ECG, serum biomarkers, cardiac imaging, and endomyocardial biopsy are recommended (7). Our case points to the importance of consideration of Takotsubo cardiomyopathy as an alternative diagnosis, and the need for endomyocardial biopsy to exclude myocarditis. This approach has clinically relevant therapeutic implications, as the patient was treated with supportive beta-blockers not steroids, and short- and long-term prognostic implications.

Our case also indicates the need for further investigation of the overlap of Takotsubo cardiomyopathy and irAEs/myocarditis in the setting of ICI therapy. For example, 2 recently published case reports with Takotsubo cardiomyopathy presentation differed in final diagnosis and treatment: 1) a patient with melanoma on ipilimumab (anti-CTLA-4) who presented with apical ballooning on TTE and focal 18F-fludeoxyglucose uptake in the cardiac apex on positron emission tomography scan was treated with beta-blockers for Takotsubo cardiomyopathy (8); whereas 2) a patient with hepatocellular carcinoma who presented with mid and apical ballooning after the first dose of nivolumab was treated with high-dose steroids for myocarditis (9). In a recent review of the WHO global database of safety reports (VigiBase), 13 cases of Takotsubo cardiomyopathy were identified among patients receiving ICIs (10). In our patient, endomyocardial biopsy was able to differentiate myocarditis by the absence of T cell infiltrate, immunoglobulin G deposition, or PD-1 expression and suggest Takotsubo cardiomyopathy with the presence of proinflammatory macrophages. There are limited data on the complications and outcomes of Takotsubo cardiomyopathy occurring with ICIs. Our patient developed grade 3 ICI pneumonitis, which was promptly treated with steroids. We speculate that the parenchymal opacities observed on the initial CT of the chest may have represented early ICI pneumonitis that triggered Takotsubo cardiomyopathy.

Our case also points to the need for increased awareness of ICI-related myocarditis and other irAEs among emergency room and urgent care physicians, primary care providers, and all health care providers. Early initiation of steroids in patients with high clinical suspicion for myocarditis is recommended (2,4,7). IrAEs often affect multiple organs concurrently, and the evaluation of other irAEs (pneumonitis, myositis) should be pursued in patients experiencing cardiovascular immune events and vice versa.

In summary, we report a case of Takotsubo cardiomyopathy occurring in the setting of nivolumab and ipilimumab treatment with clinical and cardiac imaging characteristics that overlapped with myocarditis. Endomyocardial biopsy confirmed absence of T cell infiltrate and identified proinflammatory macrophages as possible mediators of the association between ICI treatment and development of Takotsubo cardiomyopathy.

## Funding Support and Author Disclosures

The authors have reported that they have no relationships relevant to the contents of this paper to disclose.
